# Molecular Epidemiology and Trends in HIV-1 Transmitted Drug Resistance in Mozambique 1999–2018

**DOI:** 10.3390/v14091992

**Published:** 2022-09-09

**Authors:** Nalia Ismael, Eduan Wilkinson, Isabel Mahumane, Hernane Gemusse, Jennifer Giandhari, Adilson Bauhofer, Adolfo Vubil, Pirolita Mambo, Lavanya Singh, Nédio Mabunda, Dulce Bila, Susan Engelbrecht, Eduardo Gudo, Richard Lessells, Túlio de Oliveira

**Affiliations:** 1 Instituto Nacional de Saúde (INS), Estrada Nacional N1, Marracuene 3943, Mozambique; 2 Division of Medical Virology, Faculty of Medicine and Health Sciences, Stellenbosch University, Cape Town 8000, South Africa; 3 Centre for Epidemic Response and Innovation (CERI), School of Data Science and Computational Thinking, Stellenbosch University, Stellenbosch 7602, South Africa; 4 KwaZulu-Natal Research Innovation and Sequencing Platform (KRISP), School of Laboratory Medicine and Medical Sciences, University of KwaZulu-Natal, Durban 4000, South Africa; 5 Elizabeth Glaser Pediatric AIDS Foundation in Mozambique, Avenida Agostinho Neto, Maputo 620, Mozambique

**Keywords:** transmitted, drug, resistance, HIV, molecular epidemiology, Mozambique, mutations, genetic diversity, treatment naïve, temporal trend

## Abstract

HIV drug resistance (HIVDR) can become a public health concern, especially in low- and middle-income countries where genotypic testing for people initiating antiretroviral therapy (ART) is not available. For first-line regimens to remain effective, levels of transmitted drug resistance (TDR) need to be monitored over time. To determine the temporal trends of TDR in Mozambique, a search for studies in PubMed and sequences in GenBank was performed. Only studies covering the *pol* region that described HIVDR and genetic diversity from treatment naïve patients were included. A dataset from seven published studies and one novel unpublished study conducted between 1999 and 2018 were included. The Calibrated Population Resistance tool (CPR) and REGA HIV-1 Subtyping Tool version 3 for sequences pooled by sampling year were used to determine resistance mutations and subtypes, respectively. The prevalence of HIVDR amongst treatment-naïve individuals increased over time, reaching 14.4% in 2018. The increase was most prominent for non-nucleoside reverse transcriptase inhibitors (NNRTIs), reaching 12.7% in 2018. Subtype C was predominant in all regions, but a higher genetic variability (19% non-subtype C) was observed in the north region of Mozambique. These findings confirm a higher diversity of HIV in the north of the country and an increased prevalence of NNRTI resistance among treatment naïve individuals over time.

## 1. Introduction

In response to the HIV epidemic, antiretroviral treatment (ART) roll out has risen dramatically, with 28.2 million individuals on treatment by 2021 worldwide [1]. Although ART has substantially reduced HIV related morbidity, mortality, and transmission, HIV Drug Resistance (HIVDR) can become a problem, particularly in low- and middle-income countries (LMICs) where genotyping testing is not readily available [2,3]. Among ART naïve individuals, drug resistance may occur through Transmitted Drug Resistance (TDR) or Pretreatment HIV Drug Resistance (PDR) which may compromise the success of future first line regimens [4]. TDR occurs when an uninfected person naïve to antiretrovirals (ARVs) is infected with a resistant virus and PDR defined as resistance being detected among people initiating treatment or reinitiating first-line regimen after being exposed to ARVs [5].

Several studies describe TDR and PDR in LMICs since ART global scale up. One showed an increase in TDR during 2011 and 2015 in the South and East of Africa [6]. Another used data from sub-Saharan Africa and Latin American countries during 2016 and found high levels of PDR exceeding 10%, specifically to non-nucleoside reverse-transcriptase inhibitors (NNRTIs) [7]. Similarly, other systematic reviews from Latin America/Caribbean and South Africa [8] also reported high levels of PDR in 2016. Likewise, the World Health Organization (WHO) also outlined an increase in TDR in LMICs from 6.8% in 2010 to 10% and above in 2017 [9]. High levels of PDR in LMICs pose a great threat to HIV response. These threats can include lower viral suppression rates, a higher number of Acquired Immune System Deaths (AIDS) per year, higher HIV incidence, and higher ART costs [10].

In 2021, Mozambique had approximately 2.1 million adults and children living with HIV, with 1.5 million on ART, corresponding to an estimated coverage rate of 74% [11]. During the first year of free access to ART in 2004, low levels of TDR were reported (below 5%) [12]. As the coverage increased, intermediate levels of TDR in recently infected pregnant women in the city of Beira in 2007 and in Maputo in 2009 were observed [13]. Previous studies in Mozambique have also shown an increase in TDR, particularly for NNRTI [12,14,15], supporting the replacement of this class of ARVs in 2019 with dolutegravir (DTG), an integrase [12,14,15] inhibitor (INSTI) in the current first-line regimen backbone [16]. Although individual studies have given some insight into the levels of TDR, there is still no information about a broader trend in TDR during the expansion of ART access in Mozambique.

The United National Program on HIV/AIDS (UNAIDS) in 2014 launched the 90-90-90 goals, which have recently been updated to 95-95-95 goals. Achieving the third target for viral suppression is critical to reducing the rate of new HIV infections [17]. Efforts to prevent the emergence and transmission of resistant viruses are essential to eliminate HIV/AIDS by 2030 [18]. Unfortunately, lack of effective viral load testing facilities to monitor treatment, as well as the lack of genotypic testing that is not routinely available for clinical management in limited resource settings, can hinder the global HIV-1 targets [19,20]. Mozambique has a well-established prevention and treatment program but still faces challenges towards the 2030 targets. Data from 2021 indicates that 84% of the people living with HIV (PLHIV) are aware of their status. Eighty-one percent of those that know their status are on ART and 71% of those that know their status have their viral load suppressed [11]. These results clearly show that substantial efforts to achieve the UNAIDS 95-95-95 goal by 2030 are still required. Therefore, a well-established surveillance program to monitor HIVDR and continued access to treatment for PLHIV are vital.

Given the evidence of rising TDR in Mozambique accompanied by ART expansion and ineffective control of the emergence and transmission of drug resistance viruses, we performed a pooled analysis of available HIV-1 *pol* sequences retrieved from ARV naïve study populations previously performed in Mozambique. First to determine the estimated HIVDR trends in ART naïve populations and secondly to explore in detail the patterns of drug resistance mutations (DRMs) and genetic diversity over time.

## 2. Materials and Methods

### 2.1. Search Strategy and Selection Criteria

We conducted a pooled analysis of all the studies that were published about the genetic diversity and drug resistance of HIV-1 among ARV naïve patients in Mozambique.

To identify all related published articles, the search terms (HIV-1) AND (Mozambique) AND (Genetics) OR (Drug Resistance) in PubMed were used. The selection criteria of the study were as follows: First, we only included studies that described either HIVDR and Genetic Diversity in Mozambique based on the title and abstract. In this phase, we excluded all non-HIV, HIVDR, and HIV-1 studies, as well as workshop abstracts. Then, based on the full text revision, we only considered studies that included adults (aged > 15 years), treatment naïve participants, and those that performed Sanger sequencing on plasma or dried blood samples (DBS). Additionally, studies that were unable to extract related sequence data and the isolation source was not clearly identified (from treated or naïve participants, type of sample either breast milk or plasma) were also excluded. Furthermore, another search in GenBank with the key terms “Mozambique HIV-1 *pol*” was performed. During this search, an additional study with an available sequence dataset, not found during the PubMed search, was identified and included for analysis [21].

Sequences from the selected articles matched the GenBank data. For studies with no publicly available sequences, data was provided accordingly. Additionally, sequences from samples collected during the unpublished national pretreatment drug resistance (PDR) surveillance in 2018 from individuals diagnosed HIV-1 positive before initiating treatment were also included for analysis.

In addition, the online Quality Control program of the Los Alamos HIV sequence database (hiv.lanl.gov (accessed on 12 April 2022)) was used to perform a quality control of all sequences prior to any further downstream analyses. This tool performs a number of tests to help identify problems with the sequences, which include: (i) subtype information through the recombination identification program (RIP) [22]; (ii) BLAST information to verify if the sequence query belonged to HIV-1; (iii) phylogenetic tree of each single sequence with subtype references to confirm subtypes; (iv) phylogenetic tree of all sequences together with subtype references to look for duplicates or contaminations; (v) looks into hypermutations; and (vi) the existence of stop codons or frame shifts. For the drug resistance analysis, only sequences covering both the reverse transcriptase and protease regions were selected.

### 2.2. Pretreatment Drug Resistance (PDR) Surveillance Sample Selection, Viral Load Testing, and Sequencing

For the Pretreatment Drug Resistance (PDR) surveillance, only individuals between 15 and 59 years of age initiating treatment after receiving an HIV positive test result were included. DBSs were collected during 2018 in 25 health centers at a national level in Mozambique. Viral load quantification was determined using the COBAS Ampliprep TaqMan 96 (Roche Diagnostics, Indianapolis, IN, USA), according to the manufacturer’s instructions. Only samples with a viral load above 1000 copies/mL were further sequenced.

The NucliSens^®^ EasyMAG platform was used to extract total nucleic acids from DBS samples according to the manufacturer instructions. Amplification of the HIV-1 *pol* gene by one-step reverse transcriptase-polymerase chain reaction (RT-PCR) and nested PCR using HIV-1 Drug Resistance Genotyping Kit Module 1 (Applied Biosystems™, Austin, TX, USA) was performed. Subsequently, a 1.0% agarose gel electrophoresis to visualize the 1.08 kb expected band was performed. Only samples with visible bands were purified using ExoSAP-IT for PCR Product Clean-Up according to the manufacturer’s instructions (Thermo Fisher Scientific, Waltham, MA, USA). A sequencing reaction for all the purified PCR products using six different primer mixes provided by the HIV-1 Drug Resistance Genotyping Kit Module 1 (Applied Biosystems™, Austin, TX, USA) was performed. BigDye^®^ XTerminator™ Purification Kit (Applied Biosystems™, Bedford, MA, USA) was used to purify and remove dye-terminators from the sequencing reaction, followed by sequencing on the Genetic Analyzer ABI 3130 (Applied Biosystems, Foster City, CA, USA).

### 2.3. Sequence Analysis

The HIV-1 subtype was determined using the REGA HIV-1 subtyping tool version 3.0 [20]. According to the information available in the original publication or other information provided, sequences were distributed into three different regions: the south, central, and north. To calculate the proportions per time and regions for the overall NRTI, NNRTI, and PI associated TDR, the Calibrated Population Resistance (CPR) analysis tool version 8.1 was used [23]. The Stanford genotypic resistance interpretation (https://hivdb.stanford.edu/cpr/form/PRRT/ accessed on 27 August 2022) algorithm was used to determine resistance mutations.

### 2.4. Statistical Analysis

For TDR prevalence time trend analysis, sequences from different studies were grouped into three categorical variables (1999–2004, 2007–2010, and 2018). Sample collection dates were retrieved from the original articles or in GenBank collection date information. Sampling years were grouped according to the important time points of ARVs’ scale up in Mozambique as follows: the years 1999–2004 represented the time before ARVs were freely available (1999–2003) and the very beginning of free access to ARVs in the city of Maputo (2004) and 2007–2010 was characterized by a gradual roll out of ARVs in which health facilities in other regions of the country started to provide treatment. Furthermore, in 2018, the country faced a rapid roll-up of ARVs as a consequence of the test and treat strategy implementation. To assess any regional TDR differences for the different classes of ARVs (NNRTI, NRTI, and PI), we performed a geographic analysis of the sequences divided into three categorical variables: south, central, and north.

Data was analyzed using the R programming language [24]. For the categorical variables we calculated absolute frequencies and proportions. Comparison between proportions of categorical variables, was done using the chi-square test and Fisher’s exact test. All tests were considered statistically significant at *p* < 0.05.

### 2.5. Ethical Approval

The Institutional Bioethical Committee of the National Institute of Health in Mozambique granted scientific and ethical approval for the study with reference number 045/CIBS-INS/2020.

## 3. Results

### 3.1. Studies and Sequence Characteristics

**Figure 1:**
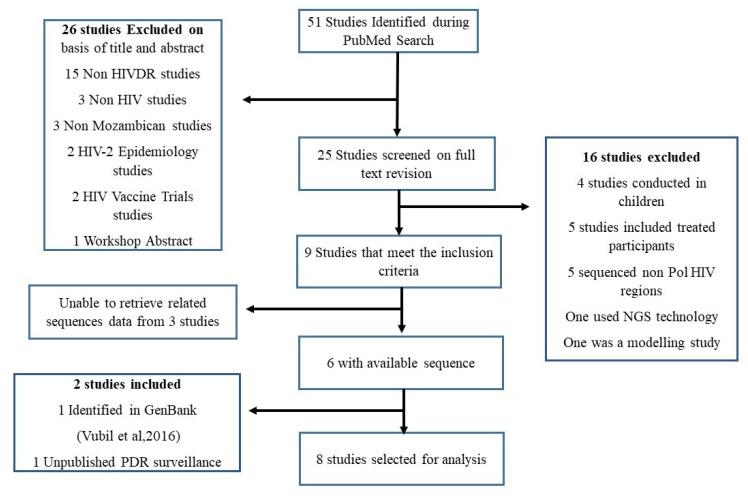
The flow diagram scheme of the literature research used to construct the dataset: NGS; Next Generation Sequencing, *pol*-Polymerase. (Vubil et al., 2016 [21]).

After searching PubMed, we initially identified 51 records. Twenty-six were excluded based on the titles and abstracts, leaving us with 25 full texts to review. Following full text revisions, 16 studies were excluded because four included children, five treated patients, another five sequenced other genes such as gp41, LTR, one used next generation technology, and one was a modeling study. The remaining nine met our inclusion criteria, but we were unable to retrieve related sequence datasets from three studies. After excluding all the studies from which we were unable to retrieve related sequence datasets and did not meet our inclusion criteria, we were left with the remaining six studies for analysis [12,13,14,15,25,26,27]. While searching in GenBank with the following terms “Mozambique HIV *pol*” an additional study not found in PubMed that looked at the genetic diversity and TDR among blood donors was found and also included in our analysis [21]. In addition to the 747 sequences from the articles identified through GenBank/PubMed, an additional 118 *pol* sequences from an unpublished PDR survey were selected (Figure 1). Overall, 8 different study datasets with a total of 865 sequences from samples collected between 1999 and 2018 were used for analysis. The summary and additional demographics of the 8 studies, including the unpublished data from the PDR surveillance, are described in the Supplementary Table S1. From the search conducted, no studies describing TDR or genetic diversity between 2011 and 2017 were found. For subtype analysis, the sequences were divided into four sampling year groups: 1999–2003, 2004–2007, 2009–2010, and 2018.

QC analysis of our dataset showed that 91.3% (*n* = 790/865) of the sequences had good quality with no stop codons, frame shifts, or hypermutations and were selected for subtype analysis. The remaining 8.6% (*n* = 75/865) were not classified into a pure HIV-1 subtype but rather into a mixture of more than five different HIV-1 subtypes and were excluded for further analysis. Furthermore, all the 790 sequences were uploaded into the Stanford HIV drug resistance program and for the drug resistance analysis, only 76.2% (*n* = 602/790) of the sequences covering both the reverse transcriptase and protease regions were selected. This selection was done once the information about the codon region of HIV sequenced was available. Further details of all 790 sequences with sampling region, year, and codon region coverage can be found in the Supplementary Table S2. Details on data set construction and analysis can be found in the Supplementary flow Figure S1. Only sequences from 2002–2018 were included for the HIVDR analysis.

### 3.2. Subtype Analysis

Rega Subtyping Tool analysis revealed that 93.5% (*n* = 739/790) of the sequences belonged to subtype C and the remaining 6.5% (*n* = 51/790) to non-C HIV-1 isolates. Further analysis of only non-C subtypes showed that 3% (*n* = 24/790) belonged to subtype A1, 1.4% (*n* = 11/790) to subtype G, and 1.4% (*n* = 9/790) to subtype D. Additionally, one 37 + cpx A1 recombinant and six HIV-1 subtype mixtures that were not classified into known Circulating Recombinant Forms (CRFs), including one C/D, one A1/D/C, two A1/C, and two A1/D were also detected (Table 1). The temporal distribution showed that the proportion of non-C did not change substantially over time, and a detailed analysis of non-C HIV-1 isolates over time is shown in Table 1.

**Figure 2:**
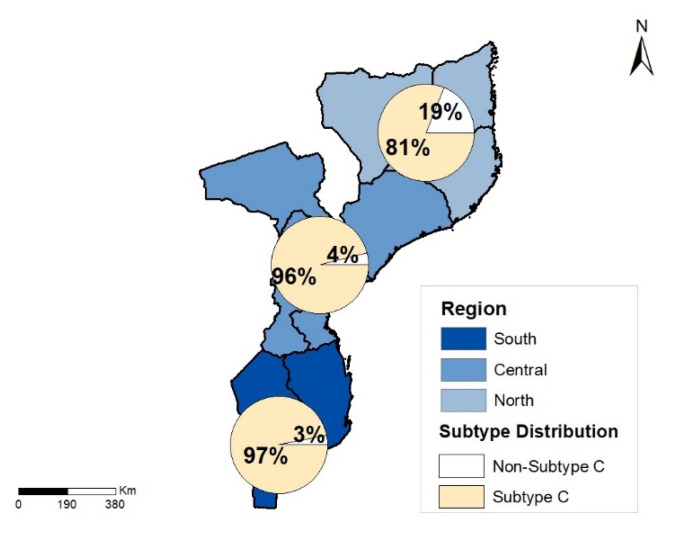
Geographic distribution of HIV-1 subtypes within the regions of Mozambique. Pie charts showing subtype C and non-C HIV-1 isolates in the north (*n* = 142), central (*n* = 270), and south (*n* = 378) of Mozambique.

To investigate the genetic diversity distribution in the country, a geographical analysis of the sequences from the south (*n* = 378), central (*n* = 270), and north (*n* = 142) regions of the country were used. For the south and central regions, 97.0% and 96% (*n* = 365 and *n* = 259) of sequences belonged to subtype C, respectively. In the north region, 81% (*n* = 115) of the sequences belonged to subtype C, while 19% (*n* = 27) belonged to non-subtype C (Figure 2).

### 3.3. Overall Changes in TDR Mutations over Time and Regions

**Figure 3:**
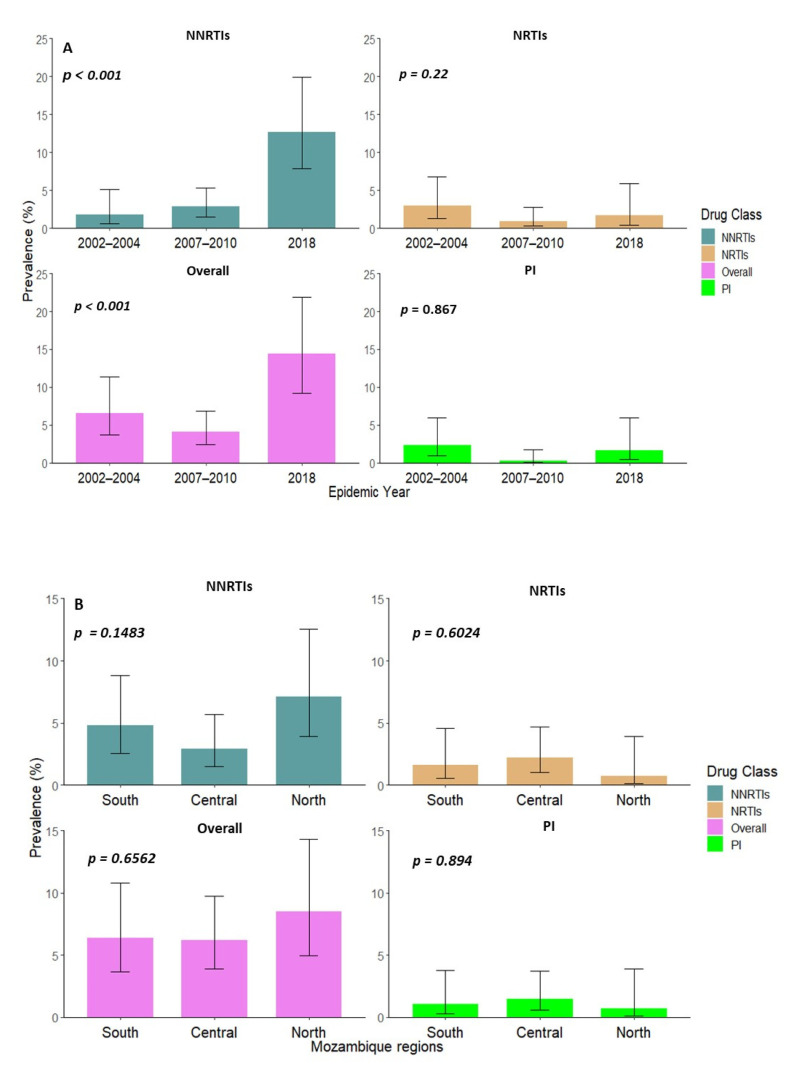
Temporal and regional trends for the overall, NNRTIs, NRTIs, and PIs having one or more major drug resistances over time in Mozambique. (**A**) Indicates the different temporal trends observed over the years. The x-axis represents the number of the HIV epidemic years since ARVs roll out, 2002–2004 (*n* = 167), 2007–2010 (*n* = 317), and 2018 (*n* = 118). (**B**) Indicates the different regions of the country. The *x*-axis represents the three different regions of Mozambique, south (*n* = 188), central (*n* = 273), and north (*n* = 141). The y-axis represents the prevalence rate of mutations calculated according to the Calibrated Population Resistance (CPR) analysis tool version 8.1. The *p*-value in each plot was established through the chi-square test over time.

From all the combined data, the overall estimated prevalence for TDR remained the same over the first two time point periods, with 6.6% (CI, 3.7–11.4%) in 2002–2004 and 4.1% (CI, 2.4–6.9%) in 2007–2009, with a statistically significant increase (*p* = 0.001) of 14.4% (CI, 9.2–21.9%) in 2018. NNRTI was the only class of ARV that showed a significant increase (*p* < 0.001) of TDR over time among ART naïve individuals, from 1.8% (CI, 0.6–5.2%) in 2002–2004 to 2.8% (CI, 1.5–5.3%) in 2007–2010, which continued increasing to 12.7% (CI, 7.9–19.9%) in 2018. For the NRTI and PI classes, the estimated TDR prevalence did not substantially change over the years and remained below 5% (Figure 3A).

No statistical difference for the TDR prevalence between the three regions for all classes of ARVs was observed (Figure 3B). For the overall resistance, the northern region showed the highest prevalence rate, with 8.5% (CI, 4.9–14.3%) when compared to the south with 6.4% (CI, 3.7–10.2%), and the central region with 3.9% (CI, 3.9–9.7%). For the NNRTI TDR prevalence rate, the north was the only region that showed a prevalence rate > 5% with 7.1% (CI, 3.9–12.6%). The estimated prevalence rate for both NRTI and PIs for all the regions were below <5% (Figure 3B).

**Figure 4:**
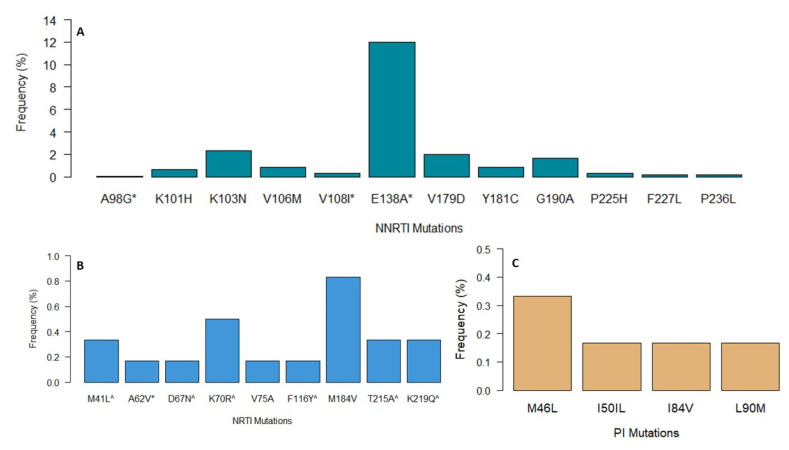
HIV-1 drug resistance mutations identified in the 602 sequences used for the analysis for the various antiretroviral drug classes (**A**) NNRTI, (**B**) NRTI, and (**C**) PIs, * polymorphic mutations (accessory mutations) and ^ Thymidine Analog Mutations (TAMs).

HIV drug resistance analysis was performed on 602 sequences covering both the reverse transcriptase (codon 1–338) and protease region (codon 1–99). Among the NNRTI mutations identified, the most common were E138A with 12.0% (*n* = 72/602), K103N with 2.3% (*n* = 14/602), followed by V179D with 2.0% (*n* = 12/602), and G190A with 1.7% (*n* = 10/602) (Figure 4). The K103N mutation causes high levels of resistance to efavirenz (EFV) and nevirapine (NVP) [28,29], which both used to be part of the previous WHO recommended first line regimen [30]. Similarly, G190A, which also causes high levels of resistance to NVP and an intermediate level of resistance to EFV [31], was also observed. E138A is a common polymorphism in HIV-1 subtype C isolates associated with low level resistance to rilpivirine (RPV) [32]. From the 72 sequences harboring this mutation, 97.2% (70/72) belonged to subtype C and the remaining 2.7% (2/72) belonged to subtypes A1 and D. Another polymorphic mutation, V179D, was also observed. It is usually selected in patients receiving EFV and can reduce susceptibility to NVP and EFV by two-fold. 

K101H is a non-polymorphic mutation selected in patients receiving NVP, EFV, and etravirine (ETR) [33] and was also identified at a low frequency (0.7%). K101H usually occurs in combination with other NNRTI-resistance mutations; alone it reduces susceptibility to NVP and EFV. NRTI mutations M184V, M41L, K70R, K219Q, and T125A were also identified at percentages below 1%. The identified M184V mutation can cause high levels of resistance to lamivudine (3TC) and emtricitabine (FTC), as well as low levels of resistance to abacavir (ABC) [34]. Of these ARVs, two (3TC and ABC) are part of the current first-line NRTI regimens recommended by WHO. Thymidine analog mutations (TAMs) that cause high-level resistance to Zidovudine (AZT) and low-level resistance to most NRTIs were also found. In total, eight and two of all patients had at least one or two TAMs, respectively. Notably, no tenofovir (TDF)-related mutations (K65R and K70E) were detected in our dataset. In total, four sequences had mutations that conferred resistance to PIs; mutation M46L was observed in two sequences; I50L and L90M in two different sequences. The I50L major mutation that causes a high level of resistance to atazanavir (ATV) and increased susceptibility to the remaining PIs was also detected in very low percentages < 1% (Figure 4).

## 4. Discussion

In this study, we explore the genetic epidemiology and TDR prevalence among ART naïve populations in Mozambique between 1999 and 2018. To obtain a broader picture of the HIV-1 genetic epidemiology, we analyzed 865 *pol* sequences reported from 8 different study datasets. Not surprisingly, subtype C predominated throughout time in Mozambique, which accounts for most infections in southern Africa [35,36,37], showing the limited need to monitor subtype distribution over time in the country. However, a higher frequency for non-C HIV-1 was observed in the north of the as previously described in other studies [12,21]. The historical relationships and intensive flux of people between the north of Mozambique and East African countries such as Tanzania and Kenya, characterized by a higher frequency of non-C HIV-1 isolates, might somehow explain this genetic profile reported in this region [38,39].

Although subtype C was the most prevalent in Mozambique, like other neighboring countries such as Tanzania, Malawi, Zimbabwe, and South Africa [40,41], we also identified pure A1, G, and D subtypes. This result suggests an epidemiological link between Mozambique and neighboring countries, which supports the impact of migratory flows of people on the spread of epidemics [42]. Furthermore, mosaic forms that may have resulted from subtype mixing but were not characterized into any known CRF/URF were observed. To better characterize the profile of these unknown mixed subtypes in Mozambique, whole genome sequencing is recommended as previously performed in South Africa where unique recombinants of subtype A1/C/D/B/K and circulating recombinants were described [43,44,45]. The presence of non-C and mixed subtypes found in our study in the north region of the country may reflect the entry of non-C subtypes from other northern frontier countries. Nevertheless, the use of next-generation sequencing (NGS) and more studies about the genetic diversity in this region are necessary to better understand this epidemiological linkage. In general, our data suggest the importance of monitoring genetic diversity using more advanced technologies and analysis to better understand the spread and transmission of non-C isolates and mixing subtypes, which can affect treatment and diagnosis as the world continues to search for an effective vaccine.

Since 2004, free access to ART has been available in Mozambique, following a rapid scale up of ARVs in 2018 when the test and treat strategy was implemented [46]. Thus, an increase in resistance levels from intermediate (5–10%) during 2002–2004 to high (14.4%) in 2018 is reported here. This same scenario is also observed in other limited resource settings, where intensive ARVs roll-outs are accompanied by an increase in TDR and as result may threaten the success of available regimens in these settings [47,48,49]. The intermediate level of resistance observed during 2002 and 2004 in our data is also comparable to the prevalence in other sub-Saharan African countries, with prevalence rates between 5% and 10% in the same time frame period before ARV rollout [49,50,51,52]. The high levels of resistance, in particular for those above 10%, result in poor population-level, outcomes especially in limited resource settings where genotypic testing is not available to monitor treatment, hindering future ARV regimens [6,53].

Not surprisingly, the increase of TDR for NNRTI over time was expected considering the low genetic barrier of this class of drugs associated with the rapid development of resistance [54]. Other factors, such as its wide use as part of the first line regimen, poor adherence support, and retention on ART, may also support the increased level of resistance reported here. As expected, common NNRTI mutations associated with high levels of resistance to EFV and NVP that composed the first line prevention of mother-to-child transmission (pMTCT) regimens widely used since 2004 in Mozambique [16,55] were also observed. A meta-analysis conducted in ART naive patients from South Africa between 2000 and 2016 from 6000 HIV-1 sequences shows that the estimated prevalence rate remained <5% until 2011 but increased to 10% in 2014 for NNRTs [56]. Our findings are consistent with other meta-analyses conducted across Africa where intermediate to high levels of TDR were also observed over time, especially for NNRTIs [57]. Additionally, high levels of resistance to NNRTI in the last decade as a preferred first-line regimen (in combination with an NRTI backbone) support the shift from NNRTI to INSTI.

On the other hand, low levels (<5%) of TDR resistance for NRTI in Mozambique over time were observed in this study. Our analysis shows that the highest TDR rate for NRTIs was observed in 2002–2004 when ART was still limited during this time. Although <5%, the emergence of such resistance strains may be explained by the inefficient and unregulated use of ARVs as well as the long use of available ARVs from other countries in the beginning before rollout.

Our study specifically shows that the most common mutation for the NNRTI (K103N) selected by EFV and NVP is also observed in other countries from the southern region of Africa [35,36,48,52,58]. This mutation is selected by EFV and NVP, and usually viruses with the K103N mutation have transmission fitness like wild-type viruses [59] which can persist for many years in the infected host. Therefore, this can explain the high prevalence of this mutation associated with frequent transmission. Clearly, a high frequency of E138A mutations was observed in our dataset, most commonly found in subtype C [32]. A study showed that this mutation in particular decreases RPV susceptibility by 2.9-fold in subtype C isolates [60]. Fortunately, Mozambique has implemented TDF PrEP instead of RPV based, supporting this choice of regimen where subtype C infections dominate in the country.

Similarly, to other studies in the same region [61,62], the M184V mutation, most common in NRTIs, was also reported here. This mutation is associated with high levels of resistance to 3TC and low levels of resistance to ABC, which are part of the first-line regimen backbone. Some TAMs were also reported, with T215A being the most frequent, followed by M41L and D67N. TAMs are usually selected when using AZT and d4T, which can be explained by the fact that these ARVs have been widely used for the first line regimen and pMTCT in Mozambique since 2004. No mutations that confer high level resistance to tenofovir (TDF) were observed. These results suggest that TDF as part of the first line regimen backbone and TDF as part of the PrEP currently implemented in Mozambique in 2021 for treatment and prevention may be effective. Most importantly, as PrEP access increases, it is important to closely monitor mutations that can further diminish PrEP efficacy.

This analysis showed low levels of resistance (<5%) for PIs throughout the different time points. A plausible explanation for this could be the high genetic barrier for this class of ARVs [63], which is also most likely associated with its little use in Mozambique. Three major PI mutations, I50L, L90M, and M46L, were found at very low frequencies. Even though low levels of resistance for PIs were observed, mutations such as the I50L raises concerns regarding the transmission of these mutations on second-line PI-based treatment regimens. Therefore, the need for a genotyping test before changing to a second-line regime is necessary and recommended in such situations.

The restricted clinical data from the selected datasets was a major limitation. At the same time, missing information about prior exposure to ARVs and HIV disclosure status at time of enrollment may somehow bias some results because it is not possible to conclude if the TDR was associated with the transmission of a resistant virus strain. Late diagnosis of HIV infection in Mozambique is common. Lack of information about the time of infection can influence the accuracy of transmitted drug mutation results because, over time, mutations can revert to wild type or to other non-resistance isolates [64]. Another limitation that made interpreting TDR over time difficult was the use of different methodologies (TDR and PDR) in previous studies, as well as the data gap between 2010 and 2018. Furthermore, the compilation of numerous independent studies undertaken in different regions and times in our study instead of a longitudinal analysis may be a limitation to our analysis because the data is not nationally and temporally representative.

## 5. Conclusions

In summary, although a higher frequency of non-subtype C isolates in the northern region of Mozambique was observed, this study confirms that subtype C dominates the HIV-1 epidemic. Moreover, our data also showed an increase in TDR from intermediate levels to a high level after ART roll out among ART naïve populations. In particular, high levels of TDR were observed for NNRTI but resistance to NRTIs and PIs remained at low levels throughout the time of analysis. Mozambique in 2019 introduced DTG, a potent IN with a high genetic barrier and high tolerability that substitutes EFV and NVP, becoming the preferred first-line regimen in combination with NRTIs. These findings provide more information about the best-fitting first-line regimens and may improve public health HIV prevention strategies. Our analysis reinforces the importance to continually evaluate HIVDR through surveillance as the epidemic progresses. Subsequently, HIVDR studies in Mozambique (and globally) must investigate the integrase region of *pol*, to better understand the impact of the switch to DTG-based regimens.

## Figures and Tables

**Table 1:** Overall time trends for the subtype distribution between 1999 and 2018.

HIV-1 Subtypes	All		1999–2003		2004–2007		2009–2010		2018	
	*n* = 790		*n* = 190		*n* = 222		*n* = 260		*n* = 118	
	*n*	%	*n*	%	*n*	%	*n*	%	*n*	%
C	739	93.5	178	93.7	210	94.6	241	-	110	93.2
A1	24	3	5	2.6	1	0.5	10	-	8	6.8
D	9	1.1	4	2.1	1	0.5	4	-	-	-
G	11	1.4	-	-	9	4.1	2	-	-	-
Recombinant of 37_cpx, A1	1	0.1	-	-	1	0.5	-	-	-	-
Mosaic form A1, C	2	0.3	1	0.5	-	-	1	-	-	-
Mosaic form A1, D	2	0.3	-	-	-	-	2	-	-	-
Mosaic form A1, D, C	1	0.1	1	0.5	-	-	-	-	-	-
Mosaic form C, D	1	0.1	1	0.5	-	-	-	-	-	-

## Data Availability

Not applicable.

## Supplementary Materials

The following supporting information can be downloaded at: https://www.mdpi.com/article/10.3390/v14091992/s1, Table S1: Summary of all eight studies selected for further analysis. Figure S1: Flow chart showing the data set construction and data analysis workflow. Table S2: Summary details of all 790 sequences selected for analysis.

## References

[1] Global HIV & AIDS Statistics—Fact Sheet|UNAIDS. https://www.unaids.org/en/resources/fact-sheet#:~:text=People%20living%20with%20HIV%20accessing,with%20HIV%20were%20accessing%20treatment.

[2] Bertagnolio S., Jordan M.R., Giron A., Inzaule S. (2022). Epidemiology of HIV drug resistance in low- and middle-income countries and WHO global strategy to monitor its emergence. Curr. Opin. HIV AIDS.

[3] Hoffmann C.J., Mills L.A., Gallant J.E., Celentano D.D., Beyrer C. (2009). Future of HIV/AIDS Care in Low- and Middle-Income Countries. Public Health Aspects of HIV/AIDS in Low and Middle Income Countries.

[4] Larder B. (2001). Mechanisms of HIV-1 drug resistance. AIDS.

[5] Clutter D.S., Jordan M.R., Bertagnolio S., Shafer R.W. (2016). HIV-1 drug resistance and resistance testing. Infect. Genet. Evol..

[6] Gupta R.K., Jordan M.R., Sultan B.J., Hill A., Davis D.H.J., Gregson J., Sawyer A.W., Hamers R.L., Ndembi N., Pillay D. (2012). Global trends in antiretroviral resistance in treatment-naive individuals with HIV after rollout of antiretroviral treatment in resource-limited settings: A global collaborative study and meta-regression analysis. Lancet.

[7] Gupta R.K., Gregson J., Parkin N., Haile-Selassie H., Tanuri A., Andrade Forero L., Kaleebu P., Watera C., Aghokeng A., Mutenda N. (2018). HIV-1 drug resistance before initiation or re-initiation of first-line antiretroviral therapy in low-income and middle-income countries: A systematic review and meta-regression analysis. Lancet Infect. Dis..

[8] Avila-Rios S., Sued O., Rhee S.-Y., Shafer R.W., Reyes-Teran G., Ravasi G. (2016). Surveillance of HIV Transmitted Drug Resistance in Latin America and the Caribbean: A Systematic Review and Meta-Analysis. PLoS ONE.

[9] WHO Releases HIV Drug Resistance Report 2021. https://www.who.int/news/item/24-11-2021-who-releases-hiv-drug-resistance-report-2021.

[10] Eaton J.W., Johnson L.F., Salomon J.A., Bärnighausen T., Bendavid E., Bershteyn A., Bloom D.E., Cambiano V., Fraser C., Hontelez J.A.C. (2012). HIV treatment as prevention: Systematic comparison of mathematical models of the potential impact of antiretroviral therapy on HIV incidence in South Africa. PLoS Med..

[11] MISAU PNC ITS HIV/SIDA Relatórios Anuais. http://www.misau.gov.mz/index.php/relatorios-anuais.

[12] Abreu C.M., Brindeiro P.A., Martins A.N., Arruda M.B., Bule E., Stakteas S., Tanuri A., de Moraes Brindeiro R. (2008). Genotypic and phenotypic characterization of human immunodeficiency virus type 1 isolates circulating in pregnant women from Mozambique. Arch. Virol..

[13] Bila D.C.A., Young P., Merks H., Vubil A.S., Mahomed M., Augusto A., Abreu C.M., Mabunda N.J., Brooks J.I., Tanuri A. (2013). Evolution of primary HIV drug resistance in a subtype C dominated epidemic in Mozambique. PLoS ONE.

[14] Bártolo I., Casanovas J., Bastos R., Rocha C., Abecasis A.B., Folgosa E., Mondlane J., Manuel R., Taveira N. (2009). HIV-1 genetic diversity and transmitted drug resistance in health care settings in Maputo, Mozambique. J. Acquir. Immune Defic. Syndr..

[15] Bellocchi M.C., Forbici F., Palombi L., Gori C., Coelho E., Svicher V., D’Arrigo R., Emberti-Gialloreti L., Ceffa S., Erba F. (2005). Subtype analysis and mutations to antiviral drugs in HIV-1-infected patients from Mozambique before initiation of antiretroviral therapy: Results from the DREAM programme. J. Med. Virol..

[16] http://www.misau.gov.mz/index.php/guioes-de-cuidados-e-tratamento?download=81:tratamento-antiretroviral-e-infeccoes-oportunistas-no-adulto-adolescente-gravida-e-crianca.

[17] Heath K., Levi J., Hill A. (2021). The Joint United Nations Programme on HIV/AIDS 95-95-95 targets: Worldwide clinical and cost benefits of generic manufacture. AIDS.

[18] Bain L.E., Nkoke C., Noubiap J.J.N. (2017). UNAIDS 90-90-90 targets to end the AIDS epidemic by 2020 are not realistic: Comment on “Can the UNAIDS 90-90-90 target be achieved? A systematic analysis of national HIV treatment cascades”. BMJ Glob. Health.

[19] Eichenberger A., Weisser M., Battegay M. (2019). HIV in Sub-Saharan Africa: Where Are We Today?. Praxis (Bern 1994).

[20] Pineda-Peña A.-C., Faria N.R., Imbrechts S., Libin P., Abecasis A.B., Deforche K., Gómez-López A., Camacho R.J., de Oliveira T., Vandamme A.-M. (2013). Automated subtyping of HIV-1 genetic sequences for clinical and surveillance purposes: Performance evaluation of the new REGA version 3 and seven other tools. Infect. Genet. Evol..

[21] Vubil A., Mabunda N., Ismael N., Ramalho D., Morgado M.G., Couto-Fernandez J.C. (2016). Genetic Diversity and Transmitted Drug Resistance of HIV-1 Subtypes in Blood Donors Candidates in Northern Mozambique. J. AIDS Clin. Res..

[22] Siepel A.C., Halpern A.L., Macken C., Korber B.T. (1995). A computer program designed to screen rapidly for HIV type 1 intersubtype recombinant sequences. AIDS Res. Hum. Retrovir..

[23] Gifford R.J., Liu T.F., Rhee S.-Y., Kiuchi M., Hue S., Pillay D., Shafer R.W. (2009). The calibrated population resistance tool: Standardized genotypic estimation of transmitted HIV-1 drug resistance. Bioinformatics.

[24] rOpenSci|How to Cite R and R Packages. https://ropensci.org/blog/2021/11/16/how-to-cite-r-and-r-packages/.

[25] Bártolo I., Zakovic S., Martin F., Palladino C., Carvalho P., Camacho R., Thamm S., Clemente S., Taveira N. (2014). HIV-1 diversity, transmission dynamics and primary drug resistance in Angola. PLoS ONE.

[26] Parreira R., Piedade J., Domingues A., Lobão D., Santos M., Venenno T., Baptista J.L., Mussa S.A.S., Barreto A.T.L., Baptista A.J. (2006). Genetic characterization of human immunodeficiency virus type 1 from Beira, Mozambique. Microbes Infect..

[27] Lahuerta M., Aparicio E., Bardaji A., Marco S., Sacarlal J., Mandomando I., Alonso P., Martinez M.A., Menendez C., Naniche D. (2008). Rapid spread and genetic diversification of HIV type 1 subtype C in a rural area of southern Mozambique. AIDS Res. Hum. Retrovir..

[28] Bacheler L.T., Anton E.D., Kudish P., Baker D., Bunville J., Krakowski K., Bolling L., Aujay M., Wang X.V., Ellis D. (2000). Human immunodeficiency virus type 1 mutations selected in patients failing efavirenz combination therapy. Antimicrob. Agents Chemother..

[29] Reuman E.C., Rhee S.-Y., Holmes S.P., Shafer R.W. (2010). Constrained patterns of covariation and clustering of HIV-1 non-nucleoside reverse transcriptase inhibitor resistance mutations. J. Antimicrob. Chemother..

[30] MISAU PNC ITS HIV/SIDA Guiões de Cuidados e Tratamento. https://www.misau.gov.mz/index.php/guioes-de-prevencao-e-de-cuidados-e-tratamento.

[31] Huang W., Gamarnik A., Limoli K., Petropoulos C.J., Whitcomb J.M. (2003). Amino acid substitutions at position 190 of human immunodeficiency virus type 1 reverse transcriptase increase susceptibility to delavirdine and impair virus replication. J. Virol..

[32] Sluis-Cremer N., Jordan M.R., Huber K., Wallis C.L., Bertagnolio S., Mellors J.W., Parkin N.T., Harrigan P.R. (2014). E138A in HIV-1 reverse transcriptase is more common in subtype C than B: Implications for rilpivirine use in resource-limited settings. Antivir. Res..

[33] Larder B.A., Kellam P., Kemp S.D. (1993). Convergent combination therapy can select viable multidrug-resistant HIV-1 in vitro. Nature.

[34] Melikian G.L., Rhee S.-Y., Taylor J., Fessel W.J., Kaufman D., Towner W., Troia-Cancio P.V., Zolopa A., Robbins G.K., Kagan R. (2012). Standardized comparison of the relative impacts of HIV-1 reverse transcriptase (RT) mutations on nucleoside RT inhibitor susceptibility. Antimicrob. Agents Chemother..

[35] Neuhann F., de Forest A., Heger E., Nhlema A., Scheller C., Kaiser R., Steffen H.M., Tweya H., Fätkenheuer G., Phiri S. (2020). Pretreatment resistance mutations and treatment outcomes in adults living with HIV-1: A cohort study in urban Malawi. AIDS Res. Ther..

[36] Hosseinipour M.C., Gupta R.K., Van Zyl G., Eron J.J., Nachega J.B. (2013). Emergence of HIV drug resistance during first- and second-line antiretroviral therapy in resource-limited settings. J. Infect. Dis..

[37] Bredell H., Martin D.P., Van Harmelen J., Varsani A., Sheppard H.W., Donovan R., Gray C.M., Williamson C., HIVNET028 Study Team (2007). HIV type 1 subtype C gag and nef diversity in Southern Africa. AIDS Res. Hum. Retrovir..

[38] Kiwelu I.E., Novitsky V., Margolin L., Baca J., Manongi R., Sam N., Shao J., McLane M.F., Kapiga S.H., Essex M. (2013). Frequent intra-subtype recombination among HIV-1 circulating in Tanzania. PLoS ONE.

[39] Kageha S., Lihana R.W., Okoth V., Mwau M., Okoth F.A., Songok E.M., Ngaira J.M., Khamadi S.A. (2012). HIV type 1 subtype surveillance in central Kenya. AIDS Res. Hum. Retrovir..

[40] Lihana R.W., Ssemwanga D., Abimiku A., Ndembi N. (2012). Update on HIV-1 diversity in Africa: A decade in review. AIDS Rev..

[41] Masoud S., Kamori D., Barabona G., Mahiti M., Sunguya B., Lyamuya E., Ueno T. (2020). Circulating HIV-1 Integrase Genotypes in Tanzania: Implication on the Introduction of Integrase Inhibitors-Based Antiretroviral Therapy Regimen. AIDS Res. Hum. Retrovir..

[42] Msimanga P.W., Vardas E., Engelbrecht S. (2015). HIV-1 diversity in an antiretroviral treatment naïve cohort from Bushbuckridge, Mpumalanga Province, South Africa. Virol. J..

[43] Adeniyi O.V., Obi C.L., Ter Goon D., Iweriebor B., Chitha W., Okoh A. (2021). Genetic Characterization of HIV-1 Subtype A1/C/D/B/K Unique Recombinant Form from Eastern Cape, South Africa. AIDS Res. Hum. Retrovir..

[44] Carr J.K., Salminen M.O., Albert J., Sanders-Buell E., Gotte D., Birx D.L., McCutchan F.E. (1998). Full genome sequences of human immunodeficiency virus type 1 subtypes G and A/G intersubtype recombinants. Virology.

[45] Wilkinson E., Holzmayer V., Jacobs G.B., de Oliveira T., Brennan C.A., Hackett J., van Rensburg E.J., Engelbrecht S. (2015). Sequencing and phylogenetic analysis of near full-length HIV-1 subtypes A, B, G and unique recombinant AC and AD viral strains identified in South Africa. AIDS Res. Hum. Retrovir..

[46] http://www.misau.gov.mz/index.php/directrizes-nacionais?download=75:directriz-de-implementacao-de-carga-viral-de-hiv-em-mocambique.

[47] Bennett D.E., Camacho R.J., Otelea D., Kuritzkes D.R., Fleury H., Kiuchi M., Heneine W., Kantor R., Jordan M.R., Schapiro J.M. (2009). Drug resistance mutations for surveillance of transmitted HIV-1 drug-resistance: 2009 update. PLoS ONE.

[48] Tshabalala M., Manasa J., Zijenah L.S., Rusakaniko S., Kadzirange G., Mucheche M., Kassaye S., Johnston E., Katzenstein D. (2011). Surveillance of transmitted antiretroviral drug resistance among HIV-1 infected women attending antenatal clinics in Chitungwiza, Zimbabwe. PLoS ONE.

[49] Arimide D.A., Abebe A., Kebede Y., Adugna F., Tilahun T., Kassa D., Assefa Y., Balcha T.T., Björkman P., Medstrand P. (2018). HIV-genetic diversity and drug resistance transmission clusters in Gondar, Northern Ethiopia, 2003–2013. PLoS ONE.

[50] Bansode V., Drebert Z.J., Travers S.A.A., Banda E., Molesworth A., Crampin A., Ngwira B., French N., Glynn J.R., McCormack G.P. (2011). Drug resistance mutations in drug-naive HIV type 1 subtype C-infected individuals from rural Malawi. AIDS Res. Hum. Retrovir..

[51] Mungati M., Mhangara M., Gonese E., Mugurungi O., Dzangare J., Ngwende S., Musasa P., Wellington M., Shambira G., Apollo T. (2016). Pre-treatment drug resistance among patients initiating antiretroviral therapy (ART) in Zimbabwe: 2008–2010. BMC Res. Notes.

[52] Hunt G.M., Ledwaba J., Kalimashe M., Salimo A., Cibane S., Singh B., Puren A., Dean N.E., Morris L., Jordan M.R. (2019). Provincial and national prevalence estimates of transmitted HIV-1 drug resistance in South Africa measured using two WHO-recommended methods. Antivir. Ther..

[53] Hamers R.L., Schuurman R., Sigaloff K.C.E., Wallis C.L., Kityo C., Siwale M., Mandaliya K., Ive P., Botes M.E., Wellington M. (2012). PharmAccess African Studies to Evaluate Resistance (PASER) Investigators Effect of pretreatment HIV-1 drug resistance on immunological, virological, and drug-resistance outcomes of first-line antiretroviral treatment in sub-Saharan Africa: A multicentre cohort study. Lancet Infect. Dis..

[54] Luber A.D. (2005). Genetic barriers to resistance and impact on clinical response. J. Int. AIDS Soc..

[55] Rupérez M., Pou C., Maculuve S., Cedeño S., Luis L., Rodríguez J., Letang E., Moltó J., Macete E., Clotet B. (2015). Determinants of virological failure and antiretroviral drug resistance in Mozambique. J. Antimicrob. Chemother..

[56] Chimukangara B., Lessells R.J., Rhee S.-Y., Giandhari J., Kharsany A.B.M., Naidoo K., Lewis L., Cawood C., Khanyile D., Ayalew K.A. (2019). Trends in Pretreatment HIV-1 Drug Resistance in Antiretroviral Therapy-naive Adults in South Africa, 2000–2016: A Pooled Sequence Analysis. EClinicalMedicine.

[57] Rhee S.-Y., Blanco J.L., Jordan M.R., Taylor J., Lemey P., Varghese V., Hamers R.L., Bertagnolio S., Rinke de Wit T.F., Aghokeng A.F. (2015). Geographic and temporal trends in the molecular epidemiology and genetic mechanisms of transmitted HIV-1 drug resistance: An individual-patient- and sequence-level meta-analysis. PLoS Med..

[58] Chimukangara B., Kharsany A.B.M., Lessells R.J., Naidoo K., Rhee S.-Y., Manasa J., Gräf T., Lewis L., Cawood C., Khanyile D. (2019). Moderate-to-High Levels of Pretreatment HIV Drug Resistance in KwaZulu-Natal Province, South Africa. AIDS Res. Hum. Retrovir..

[59] Wertheim J.O., Oster A.M., Johnson J.A., Switzer W.M., Saduvala N., Hernandez A.L., Hall H.I., Heneine W. (2017). Transmission fitness of drug-resistant HIV revealed in a surveillance system transmission network. Virus Evol..

[60] Tambuyzer L., Nijs S., Daems B., Picchio G., Vingerhoets J. (2011). Effect of mutations at position E138 in HIV-1 reverse transcriptase on phenotypic susceptibility and virologic response to etravirine. J. Acquir. Immune Defic. Syndr..

[61] Xu H.-T., Colby-Germinario S.P., Asahchop E.L., Oliveira M., McCallum M., Schader S.M., Han Y., Quan Y., Sarafianos S.G., Wainberg M.A. (2013). Effect of mutations at position E138 in HIV-1 reverse transcriptase and their interactions with the M184I mutation on defining patterns of resistance to nonnucleoside reverse transcriptase inhibitors rilpivirine and etravirine. Antimicrob. Agents Chemother..

[62] Hunt G.M., Ledwaba J., Basson A.E., Moyes J., Cohen C., Singh B., Bertagnolio S., Jordan M.R., Puren A., Morris L. (2012). Surveillance of transmitted HIV-1 drug resistance in Gauteng and KwaZulu-Natal Provinces, South Africa, 2005–2009. Clin. Infect. Dis..

[63] Price M.A., Wallis C.L., Lakhi S., Karita E., Kamali A., Anzala O., Sanders E.J., Bekker L.-G., Twesigye R., Hunter E. (2011). IAVI Early Infection Cohort Study Group Transmitted HIV type 1 drug resistance among individuals with recent HIV infection in East and Southern Africa. AIDS Res. Hum. Retrovir..

[64] Dandache S., Coburn C.A., Oliveira M., Allison T.J., Holloway M.K., Wu J.J., Stranix B.R., Panchal C., Wainberg M.A., Vacca J.P. (2008). PL-100, a novel HIV-1 protease inhibitor displaying a high genetic barrier to resistance: An in vitro selection study. J. Med. Virol..

