# Supplementary material for: Molecular Epidemiology and Trends in HIV-1 Transmitted Drug Resistance in Mozambique 1999–2018
Source: Viruses. 2022 Sep 9;14(9):1992. doi: 10.3390/v14091992 (PMC9505726; doi:10.3390/v14091992)
Supplement: Supplementary file 1 [file viruses-14-01992-s001.zip › Supporting Information Figure S1.pdf]

Supporting Information: Figure S1 Flow chart showing the data set construction and data analysis workflow

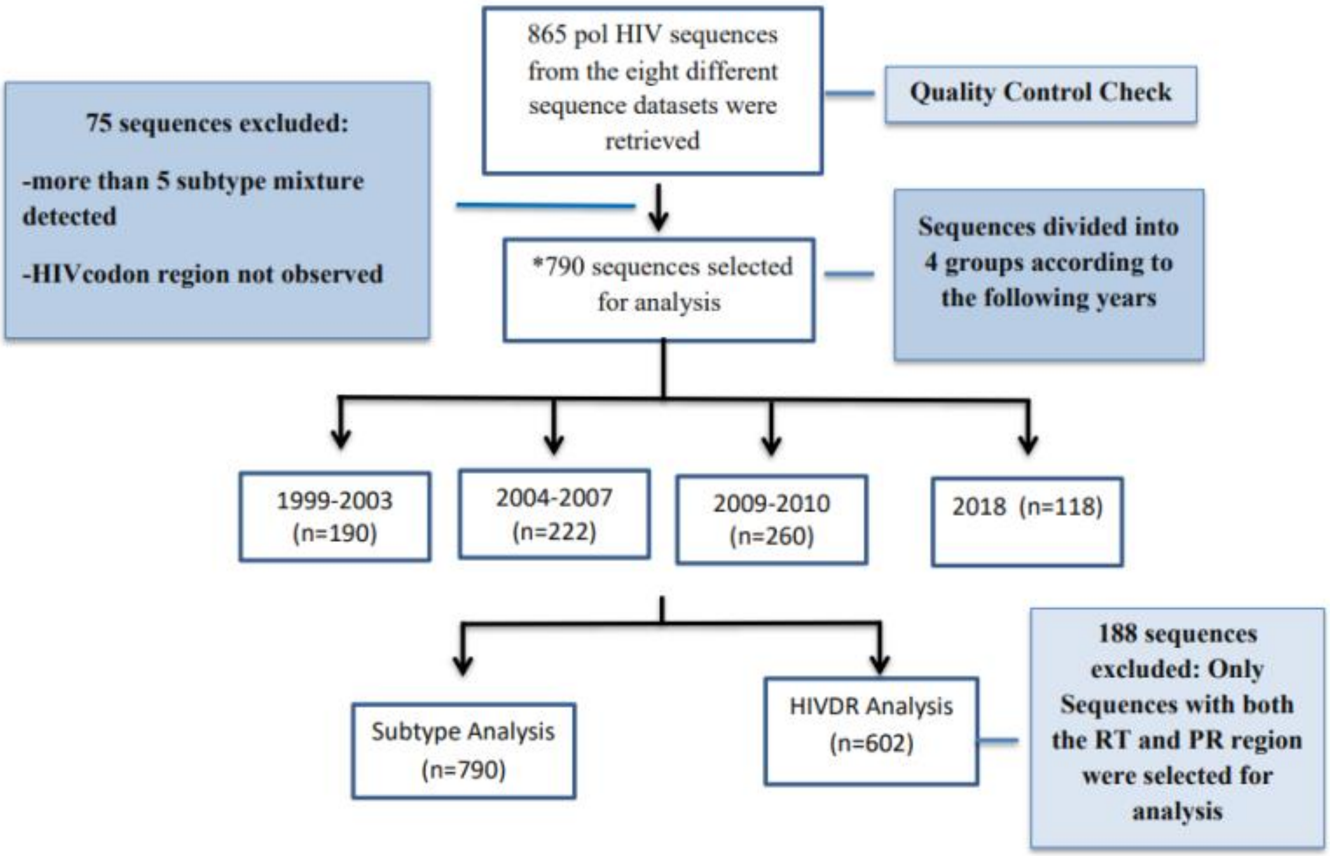

* Year of Sampling was further confirmed by GenBank Information or full text description of published studies

HIVDR- HIV Drug Resistance ,PR-Protease , RT-Reverse Transcriptase
